# Sociodemographic inequalities in the epidemiology and vaccine uptake within a large outbreak of measles in Birmingham, England, 2023 to 2024

**DOI:** 10.2807/1560-7917.ES.2025.30.16.2400652

**Published:** 2025-04-24

**Authors:** Hannah Jary, Adam Pullen, David Howett, Erjola Hani, Shakeel Suleman, Lisa Byrne, Emma Booth, Richard Puleston, Vanessa Saliba, Colin NJ Campbell, Carol Chatt

**Affiliations:** 1 Field Services Midlands, UK Health Security Agency, Birmingham, United Kingdom; 2 UK Field Epidemiology Training Programme, Field Services, UK Health Security Agency, London, United Kingdom; 3 Immunisation Programmes Clinical Policy & Implementation Division, UK Health Security Agency, London, United Kingdom; 4 Acute Respiratory Unit, UK Health Security Agency, London, United Kingdom; 5 Field Services Rapid Investigation Team, UK Health Security Agency, London, United Kingdom; 6 West Midlands Health Protection Team, UK Health Security Agency, Birmingham, United Kingdom; * These authors contributed equally to this work and share first authorship.

**Keywords:** measles, outbreak, inequalities, MMR, vaccination, ethnicity, deprivation

## Abstract

Measles disproportionately affects under-vaccinated communities, and inequalities in vaccination coverage exist in the United Kingdom (UK). In Birmingham, England, 406 confirmed measles cases were notified to the UK Health Security Agency between 13 October 2023 and 12 April 2024. Public health case management system data and primary care vaccination data were used to describe the epidemiology of the outbreak. Cases had a median age of 5.5 years (interquartile range (IQR): 1–13); 53% (214/406) were male, 45% (183/406) female and sex was unknown for 2% (9/406). Most cases (89%; 362/406) were unvaccinated. While 78% (315/406) of cases occurred in the city’s most deprived areas (quintile 1), none did in the least deprived. The measles rate per 100,000 was 47.6 in quintile 1 vs 13.8 in quintile 3. Across ethnicities, the rate was 86.3 in Black African vs 10.8 in White British. Increases in vaccination rates between the outbreak period and an equivalent prior non-outbreak period seemed higher in most deprived populations (0.5% in quintile 1 vs 0.3% in quintiles 5). Variations, however, were observed between ethnic groups. In this large outbreak, measles disproportionately affected individuals from socioeconomically deprived backgrounds and ethnic minorities. In underserved communities, continued tailored services and vaccinations are required.

**Key public health message**

**What did you want to address in this study and why?**

We wanted to describe a large measles outbreak in Birmingham, the second largest city in England, which affected more than 400 people, most of whom were unvaccinated against measles virus. We also wanted to highlight inequalities between socioeconomic and ethnic groups with regards to the risk of measles and to examine if the uptake of the vaccination offer, delivered as part of the outbreak response, differed between these groups.

**What have we learnt from this study?**

Deprived communities and some ethnic groups were disproportionately impacted by the measles outbreak. The measles–mumps–rubella (MMR) vaccination catch-up campaign was effective at targeting the most deprived populations, who were at highest risk of measles. However, variations in MMR coverage gain occurred across ethnicities, with only small increases in MMR uptake in some ethnic groups that were most impacted by the outbreak.

**What are the implications of your findings for public health?**

Inequalities in vaccination coverage in England exist and the risk of large outbreaks remains. Understanding the differences in measles risk and MMR uptake between different sociodemographic groups is essential to drive local public health interventions and implementation of tailored vaccination services for underserved communities. This is essential to protect vulnerable groups and the wider population.

## Background

Measles is a highly contagious, airborne virus which spreads from person to person by respiratory droplets: one infected person can infect nine of 10 of their non-immunised close contacts [1]. Cases typically present with cough, coryzal symptoms, conjunctivitis, high fever (≥ 39°C) and an erythematous maculopapular rash, and complications include encephalitis, blindness, pneumonia, and death. Measles is preventable with a safe and effective vaccine. In the United Kingdom (UK), the routine immunisation schedule offers two doses of the measles–mumps–rubella (MMR) vaccine. The first to individuals aged 12 months and older, and a subsequent booster to those aged 3 years 4 months or older.

In 2023, there was a global resurgence of measles and the World Health Organization’s (WHO) European Region reported an alarming 30-fold increase in measles cases [2]. This sharp rise in cases was attributed to falling vaccination rates in children, compounded by the effect of the COVID-19 pandemic [2].

The UK had been declared by the WHO to have eliminated measles in 2017, but this status was not maintained following a marked increase in cases in 2018. During the COVID-19 pandemic, measles transmission was interrupted with the UK re-gaining elimination status in 2021 [3]. However, in 2023, increasing measles activity was noted in the UK, in keeping with the global context [4,5]. Vaccine coverage has declined between 2014 and 2024, and population susceptibility estimates for all birth cohorts have shown England could sustain disease transmission in particular age cohorts (e.g. people born between 1998–1999 and 2003–2004) and areas of low coverage (e.g. London) [5-7].

Inequalities in vaccination uptake in the UK have been well documented [8-10] and under-vaccinated communities are disproportionately affected by measles outbreaks [11-15]. Examples of such communities include some ethnic groups and recent migrants, as well as Charedi Orthodox Jewish communities, anthroposophical communities (e.g. Steiner schools) and people identifying as belonging to ‘Gypsy and Irish Traveller’ communities according to the Office of National Statistics (ONS) classification [16]. A health equity audit of the national immunisation programme for England published in 2021 concluded that while overall vaccine coverage for routine immunisations in the country is high, there are still avoidable inequalities in vaccination within some population groups [17].

The UK Health Security Agency (UKHSA) is committed to tackling health inequalities by identifying people with the highest risk of hazards to health, including infectious diseases, and protecting these groups with tailored approaches [18]. The UKHSA Immunisation Inequalities Strategy recognises that there are complex reasons behind vaccine coverage inequalities. In keeping with the WHO’s Tailoring Immunisation Programmes approach, the strategy advises that to reduce coverage inequalities, locally designed, multi-component interventions addressing barriers to vaccine uptake can be effective [19]. This approach aims to integrate people-centred research and behavioural insights into immunisation programmes to develop solutions which support, motivate and enable people from all population groups to be vaccinated [20].

Birmingham, the UK’s second largest city, has an ethnically diverse population. In the 2021 Census, 43% of the population identified as ‘White British’, 31% as combined ‘Asian’ ethnicities and 11% as combined ‘Black’ ethnicities [16]. There are high levels of deprivation in Birmingham, with 51% of under 16-year-olds living in the 10% most deprived areas in England [21]. Information from the national Cover of Vaccination Evaluated Rapidly programme (COVER), which uses data from the National Health Service (NHS) Child Health Information System (CHIS), indicates that 82.1% of children in Birmingham had received their first dose of MMR by their second birthday and 75.1% had received their second dose by their fifth birthday in 2022–2023, lower than the England rates of 89.3% and 84.5%, respectively [6]. Despite increasing measles cases across Europe and declining vaccination rates in the UK, Birmingham experienced an almost 11-month absence of confirmed measles cases before the index case of the outbreak described here.

## Outbreak detection

On 13 October 2023, UKHSA was notified that two confirmed measles cases (London residents) had stayed with relatives in Birmingham and visited a Birmingham hospital while infectious. UKHSA conducted contact tracing in Birmingham and identified two unvaccinated individuals who had developed symptoms (fever, cough and, subsequently, rash) after contact with the confirmed cases: an adult and a young child (under 5 years of age) in the same family. The adult subsequently tested positive, becoming the first known confirmed case in a Birmingham resident in 2023. The child was considered a probable case due to their epidemiological link, symptoms and non-immune status, but confirmatory testing was not completed (the oral fluid test kit was not returned in the post). Subsequently, six additional cases were confirmed among Birmingham residents (from different parts of the city compared with the first affected family) with symptom onset in the first 2 weeks of November 2023: all were unvaccinated children. Two had no known exposure (primary cases), two were siblings of a primary case, one had a known healthcare contact with a confirmed case, and one had no exposure information recorded. By December, UKHSA were responding to outbreaks in six schools across the city (often linked by family members) and a sharp increase in case numbers suggested that community transmission had become established in some parts of the city.

Here, we report the epidemiological description of cases from the first 6 months of this large measles outbreak in Birmingham, with a focus on the sociodemographic inequalities within the outbreak. In keeping with UKHSA’s Immunisation Inequalities Strategy, we describe inequalities in vaccination uptake, while also identifying high risk communities where control measures should be focused.

## Methods

### Surveillance, case finding and laboratory testing

In the UK, measles is a notifiable disease. Possible, probable or confirmed cases of measles are reported to UKHSA by clinicians or laboratory services via the national mandatory infectious disease notification system. A risk assessment for all possible, probable or confirmed cases is undertaken by UKHSA [22].

Oral fluid testing kits are sent by courier or post to all known possible or probable cases (and cases confirmed by local laboratory testing) which, if returned, are tested by the reference laboratory (Virus Reference Department, UKHSA, Colindale, London) for IgM and IgG (and PCR analysis for RNA detection if the sample is taken within 7 days of symptom onset). From March 2024, the testing algorithm changed: UKHSA no longer test for IgG but performs PCR testing on all IgM positive samples (where the sample is adequate). Positive clinical samples from local hospital laboratories should be forwarded on to the reference laboratory for confirmatory testing and sequencing.

### Case definition

A confirmed case was defined as a Birmingham resident with laboratory confirmation (measles IgM in blood or oral fluid in the absence of recent vaccination (within 14 days) or confirmed wildtype measles RNA in any clinical specimen), notified to UKHSA between 13 October 2023 and 12 April 2024. Cases with detection of measles IgM or measles RNA from local laboratory testing were included as confirmed cases, unless subsequent reference laboratory testing was negative in which case, the national measles team determined whether the case was discarded or retained as confirmed.

A probable case was defined as clinically classical case of measles with epidemiological features that either increase the likelihood of the patient having been exposed (such as links to under-vaccinated communities, including Charedi Orthodox Jewish communities, ‘Gypsy and Irish Traveller communities’, Anthroposophic (Steiner) communities or migrants) and/or favour the diagnosis of measles relative to other causes of rash illness, in the absence of laboratory confirmation [22].

A possible case was defined as an individual where measles is considered as a plausible cause of their illness but does not meet the definition of a probable case either because it is not clinically classical or because the epidemiological context is not suggestive of measles.

### Epidemiological investigation

Once an outbreak was declared on 7 November 2023, a line list was maintained, compiling data from the UKHSA case management system, laboratory surveillance system and immunisation records (including vaccination data from UKHSA’s Immunisation Information System (IIS), extracted from primary care immunisation records, and CHIS). The line list included information collected as part of the risk assessment undertaken with cases, including potential sources of exposure and links to educational or healthcare settings in their incubation or infectious period. The line list was enriched with ethnicity data using IIS. Index of multiple deprivation (IMD) scores were derived based on the lower super output area (a geographical area within England comprising of between 400 and 1,200 households) of the case’s residential postcode [23]. Hospitalisation data were obtained by linking to NHS Secondary Uses Service data, and cases who had been admitted as a hospital inpatient or who had attended the emergency department within 5 days before or 10 days after their date of onset were identified. Ethnicity and IMD population estimates for Birmingham from the ONS 2021 census were used for the calculation of case rates [16,24]. Age-stratified relative risks (RR) were calculated for age–ethnicity groups with case numbers greater than five, with the ‘White British’ group used as a reference group for each of the age stratum up to 18 years of age [25]. Relative risks were not adjusted for confounding factors.

Throughout the outbreak, the line list was used to provide summaries of the descriptive epidemiology of the cases across the city, to inform the outbreak response and control measures. Epidemiological links between notified cases were identified using the information collated on the line list, and where indicated, setting specific epidemiological situation reports.

### Immunisation coverage data collection and analysis

In May 2024, MMR vaccine and eligible individual records from all residents of the Birmingham upper tier local authority (ONS reference code: E08000025) who were aged between 1 and 25 years old were extracted from IIS for two separate periods. IIS was specifically chosen rather than COVER for this purpose due to its ability to curate custom cohorts, as well as its inclusion of additional sociodemographic information such as ethnicity and regional deprivation (i.e. IMD). The age range was chosen from the availability of reliable historic data in IIS. A baseline period was assessed where neither an outbreak or catch-up campaign had occurred (i.e. standard vaccination policies), covering August 2022 to April 2023 (non-outbreak period), as well as our period of interest which included MMR vaccines administered during the outbreak and a subsequent vaccine catch-up campaign between August 2023 and April 2024 (outbreak period). For each period, the proportion and number of eligible individuals vaccinated with one and two doses of MMR were calculated in August, and again in April for the same cohort identified in August, and the difference in vaccination coverage between the two periods assessed and compared. These statistics were further stratified by age, ethnicity and IMD as recorded for every individual in August of each period.

The rationale behind the date range for our period of interest was twofold. Firstly, to cover vaccines administered during the outbreak and catch-up campaign, but also to mitigate the unavailability of data between September 2023 and March 2024 following dataflow issues with IIS. The baseline period was selected to reflect our period of interest without the effects of the outbreak and catch-up campaign.

Geographical linkages were based on postcode of residence using the ONS Postcode Lookup dataset (May 2023). Ethnicity, which is self-declared by each individual, or parent or guardian for children, on signing up for primary healthcare, includes categories as set out in the ONS England and Wales Census disaggregated ethnicity classification system (2011) [26,27]. Data cleaning involved the exclusion of invalid records (Table 1).

**Table 1:** Criteria for excluding vaccine and individual records

Records	Reasons for exclusion
Vaccine records^a^	Dose number recorded as more than two
	First dose administered before 12 months of age
	Interval between doses 1 and 2 less than 15 months (dose 2 records only)
Eligible individual records	Had no eligible individual record (where vaccine record present)
	Invalid or missing general practice identifier (Organisation Data Service code)
	Had a recorded date of death
	Invalid, missing, or not a post code of residence in England

^a^ For vaccine records, these criteria were developed to remove records that did not adhere to immunisation against infectious disease guidance (https://www.gov.uk/government/collections/immunisation-against-infectious-disease-the-green-book).

### Data analysis and statistical methods

The data was retrieved using Microsoft SQL Server 2019, and analysis performed using R V.4.2.1 [28]. Chi-square tests were used for the comparison of categorical data.

## Results

### Outbreak description and outcomes

**Figure 1:**
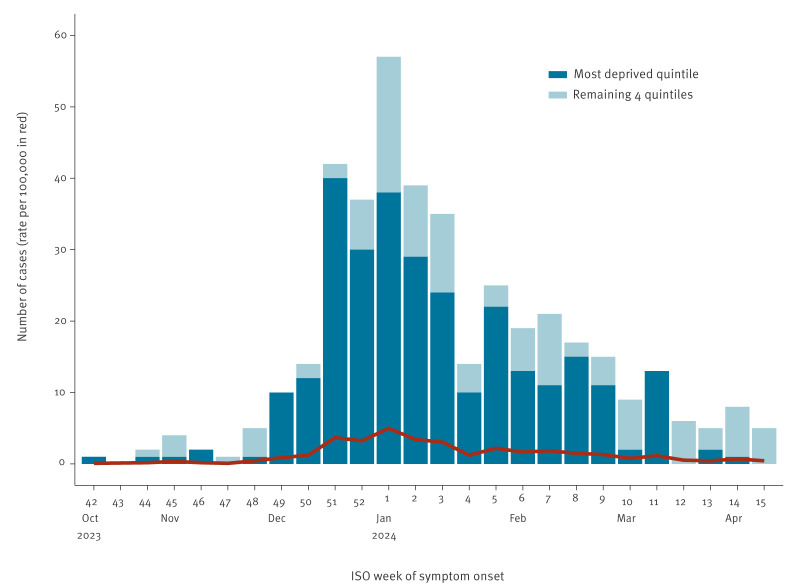
Number of confirmed measles cases and their rate per 100,000 population^a^ by ISO week of symptom onset^b^, stratified by residence in the most deprived and remaining IMD quintiles, Birmingham, United Kingdom, 13 October 2023–12 April 2024 (n = 406 cases)

IMD: index of multiple deprivation; ISO: International Organization for Standardization; ONS: Office for National Statistics.

^a^ The crude case rate per 100,000 was plotted (red line) for all confirmed cases in Birmingham residents, using ONS 2021 census denominator data [16].

^b^ Symptom onset is defined using a hierarchy of rash onset date > other symptom onset date > observed onset date (the date recorded by the Health Protection Team when symptom onset unknown) > date of entry into the line list, depending on data availability.

There were 406 confirmed cases of measles in Birmingham residents notified to UKHSA between 13 October 2023 and 12 April 2024 (Table 2). Case numbers peaked in week 1 of 2024, with 56 confirmed cases (Figure 1). Overall, the median age was 5.5 years (interquartile range (IQR): 1–13), with most cases (282/406; 69%) aged 10 years old or under. A total of 214 cases (53%) were male, 183 (45%) female and for nine (2%), information on sex was unavailable (Table 2). Cases were reported in 85 educational settings (including nurseries, schools, colleges and universities) and the number of cases per educational setting ranged from 1 to 18. Five children were known to be home schooled, and the educational setting was unknown for 34 (20%) of 170 children of school age (between 4 and 16 years-old). The outbreak continued with low case numbers until September 2024 but here we report on cases in the first 6 months.

**Table 2:** Characteristics of all confirmed cases of measles in residents, by vaccination status, Birmingham, United Kingdom, 13 October 2023–12 April 2024 (n = 406 cases)

Characteristic	Cases, n = 406		MMR vaccination doses					
			One dose, n = 28		Two doses, n = 16		Unvaccinated, n = 362	
	Number^a^	%^a^	Number^b^	%^b^	Number^b^	%^b^	Number^b^	%^b^
**Age group in years**								
< 1	56	14	0	0	0	0	56	100
1–4	124	31	11	9	1	1	112	90
5–11	111	27	9	8	2	2	100	90
12–18	54	13	6	11	1	2	47	87
≥19	61	15	2	3	12	20	47	77
**Sex**								
Female	183	45	11	6	9	5	163	89
Male	214	53	17	8	6	3	191	89
Unknown	9	2	0	0	1	11	8	89
**Ethnicity**								
Asian or Asian British	118	29	7	6	3	3	108	92
Black or Black British	97	24	9	9	1	1	87	90
Mixed	30	7	0	0	0	0	30	100
Other	24	6	1	4	0	0	23	96
Unrecorded	67	17	1	1	3	4	63	94
White	70	17	10	14	9	13	51	73
**Index of multiple deprivation**								
1 – Most deprived	315	78	20	6	11	3	284	90
2	69	17	6	9	3	4	60	87
3–5	22	5	2	9	2	9	18	82

MMR: measles–mumps–rubella vaccination; IQR: interquartile range.

^a^ Number and percentage of the overall number of cases (n = 406).

^b^ Number and percentage of the number of cases in that characteristic category.

Ethnicity and index of multiple deprivation quintiles are aggregated to avoid deductive disclosure.

Of the 342 cases with available data, 145 (42%) were admitted to hospital and a further 137 (40%) attended the emergency department without being admitted.

### Outbreak inequalities

**Figure 2:**
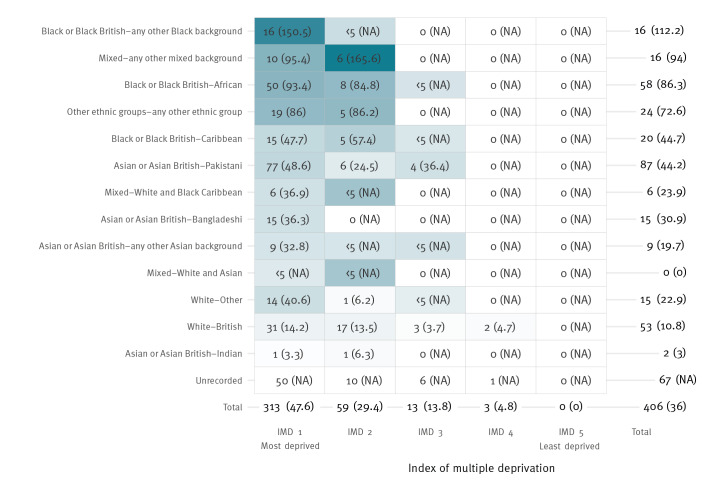
Number^a^ and rate^a^ of confirmed cases of measles in residents, by ethnicity and IMD quintile, Birmingham, United Kingdom, 13 October 2023–12 April 2024 (n = 406 cases)

IMD: index of multiple deprivation; NA: not applicable^a^.

^a^ Not applicable denotes non-calculated values. Where the population is less than 10,000, counts and totals that are less than five have been suppressed. These have also been deducted from the rates and column/row totals shown.

A total of 315 of the 406 (78%) cases resided in an LSOA categorised as being in the most deprived IMD quintile (Table 1 and Figure 1), while there were no cases among residents of LSOAs categorised as being in the least deprived quintile. The rate of measles was 47.6 per 100,000 residents in quintile 1 (most deprived) vs 29.4 and 13.8 in quintiles 2 and 3 respectively (Figure 2).

Rates per 100,000 are indicated in brackets. The colour gradient reflects the rate of confirmed cases (continuous gradient from 0 to maximum rate (165.6)).

The ethnic groups are ordered, from highest to lowest, by their true rate (data not shown), not the rate shown with suppressed cases deducted.

At the peak of the outbreak (mid-January 2024), ethnicity data using routine sources was only known for 52% (99/191) of cases. Following enrichment for the purpose of post-outbreak retrospective analysis, ethnic origin was known for 339 of 406, 83%. Of these, the most common ethnicity was ‘Asian or Asian British – Pakistani’ (87 of 339, 26%) followed by ‘Black or Black British – African’ (58 of 339, 17%) and ‘White British’ (53 of 339, 16%) (Figure 2); the case rates in these ethnic groups were 44.2, 86.3 and 10.8 per 100,000 population, respectively (Figure 2). The rate of measles was highest in the ‘Black and Black British – any other Black background’ ethnic group, with 112.2 cases per 100,000 population (Figure 2). In each ethnic group, the majority of cases were in residents of LSOAs categorised as being in the most deprived IMD quintile (IMD 1).

**Figure 3:**
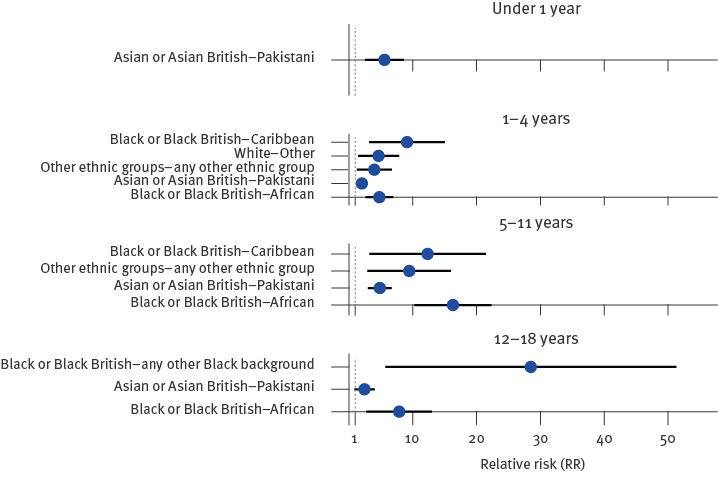
Age-stratified relative risk^a^, of confirmed cases of measles by ethnic group in residents aged 0–18 years, Birmingham, United Kingdom, 13 October 2023–12 April 2024

^a^ The ‘White British’ ethnic group has been used as the reference group for the relative risk in each age stratum. Ethnic groups with five or less cases in an age group have been excluded. The black horizontal lines represent 95% confidence intervals.

Age-stratified RRs by ethnic group (Figure 3) show that in each of the age groups up to the age of 18 years, all other ethnic groups (with more than five cases in that age group) had a higher risk of cases than the ‘White British’ group. In the ‘Asian or Asian British – Pakistani’ population, the RR was 5.6 (95% CI: 2.5–8.6), 2.0 (95% CI: 1.3–2.7), 4.8 (95% CI: 3.0–6.7) and 2.4 (95% CI: 0.8–4.0) in under 1-year-olds, 1–4-year-olds, 5–11-year-olds and 12–18-year-olds respectively. The highest RR was seen in the ‘Black or Black British – any other Black background’ population in 12–18-year-olds, who were estimated to have a 28.5 times higher risk of cases than the ‘White British’ population of the same age (RR: 28.5; 95% CI: 5.7–51.3).

### Case vaccination status 

Of the 406 cases, 56 (14%) were too young to have received their first MMR vaccination. Among the 350 confirmed cases aged over 12 months, 87% (n = 306) had no record of MMR vaccination;  of these, three were born before 1968 when the first measles containing vaccine was introduced.

Of those with known vaccination status who were old enough to have had at least one dose, only 11% (31/279) of cases residing in the most deprived quintile (IMD quintile 1) had one or two recorded doses of MMR, compared with 18% (13/71) of cases in IMD quintiles 2–4, although this difference is not statistically significant (chi-square test p = 0.10).

Of the 310 cases eligible for vaccine with known ethnicity, cases of ‘White’ ethnicity were more likely to have had at least one dose of vaccine (19 of 67, 28%) than cases of all other ethnic groups combined (21 of 243, 9%) (chi-square test p = 0.00).

## Outbreak control measures

### Health protection response

From the beginning of the outbreak, UKHSA followed up all cases to undertake a risk assessment and contact tracing, provide exclusion advice and information for cases and contacts (e.g. avoiding locations with high risk of disease transmission such as schools, healthcare settings), and assess the requirement for post-exposure prophylaxis (vaccination or immunoglobulin) for contacts [22]. Incident Management Teams were convened to respond to clusters of cases in specific settings, such as educational settings. Daily surveillance reports were developed by UKHSA, as well as individual setting level epidemiological reporting. System partnership working, including UKHSA, the NHS and local authorities, was set up across the city and the West Midlands region, with community engagement embedded in the response. A national measles incident was declared on 9 January 2024, predominantly driven by cases in Birmingham [29].

### Vaccination

In response to the outbreak, NHS England commissioned a local MMR catch-up campaign in primary care in December 2023 for children aged 1–5 years, in addition to routine primary care MMR provision. Where school outbreaks were identified, school vaccination sessions were organised and prioritised on likely benefit and capacity. MMR vaccination clinics were also offered proactively in school settings (i.e. in schools that were not part of the outbreak) during February and March 2024, for incompletely immunised children and staff. This proactive offer included 16 primary and nine secondary schools with the highest numbers and proportions of susceptible children according to CHIS data. A national catch-up campaign was launched in February 2024 for incompletely immunised children up to age 11 years; in Birmingham this was extended to age 25 years. Individuals were invited to book their MMR vaccine through their general practice.

According to IIS data for all ages (1–25 years), MMR dose 1 vaccination coverage increased by 0.6% in the non-outbreak period and by 1.1% in the outbreak period: an additional 0.5% increase in coverage (2,230 doses) was achieved in the outbreak period compared with the non-outbreak period. For MMR dose 2, the difference between the periods was 0.4% (2,963 doses). For dose 1, the largest difference between the non-outbreak and outbreak periods was seen in ages 1–5 years and ages 6–11 years (a difference of 0.8% in both groups). For dose 2, the largest difference between time periods was 0.8% in 6–11-year-olds.

**Figure 4:**
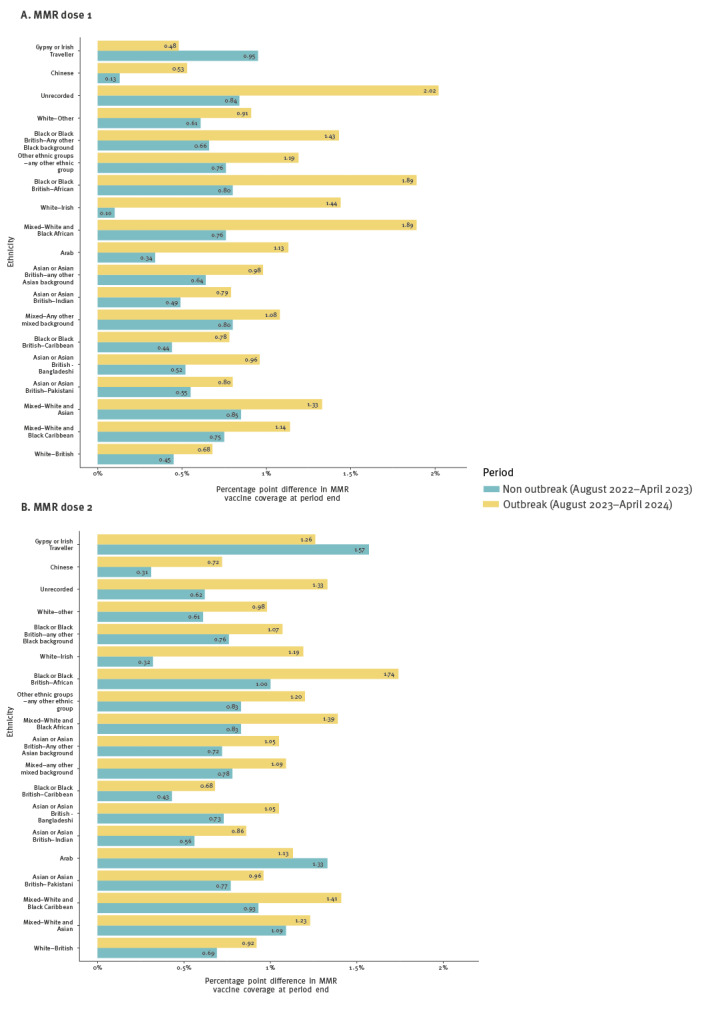
Percentage differences in MMR vaccine coverage at the end of each period compared with the start, stratified by ONS 2011 census based ethnic groups, Birmingham, United Kingdom, 13 October 2023–12 April 2024

MMR: measles–mumps–rubella vaccination; ONS: Office for National Statistics.

There was substantial variation in the difference between the change in vaccination coverage in the non-outbreak period and the outbreak period across different ethnic groups (Table 3 and Figure 4). The ethnic group with the largest difference for dose 1 was ‘White Irish’ (1.3%, a difference of 12 doses), followed by unrecorded ethnicity (1.2%, 723 doses). The ‘White British’ group had one of the lowest differences (0.2%, 261 doses), as did ‘Asian or Asian British – Pakistani’ (0.3%, 237 doses) which is one of the groups with the highest case rate in the outbreak (Table 3). ‘Black or Black British – African’, the group with the highest case rate in the outbreak, had 1.1% higher increase in coverage (348 additional doses) in the outbreak period compared with the previous period. ‘Gypsy or Irish Travellers’ were the only group to have a smaller increase in coverage in the outbreak year compared with the non-outbreak year, but the population size is very small (only two fewer doses were given in the outbreak period compared with the non-outbreak period).

**Table 3:** MMR dose 1 coverage across the non-outbreak and outbreak periods and apparent increase observed between periods, for different ethnic groups and deprivation quintiles Birmingham, United Kingdom, 13 October 2023–12 April 2024

Characteristic	MMR dose 1 uptake						Change in percentage vaccinated after campaign
	Non outbreak period (2022–2023)			Outbreak period (2023–2024)			
	Start coverage %	Increase % (number of extra doses)	End coverage %	Start coverage %	Increase% (number of extra doses)	End coverage %	
**Ethnicity**							
White British	83.13	0.45 (528)	83.58	84.58	0.68 (789)	85.26	0.23 (261)
Asian or Asian British – Pakistani	78.77	0.55 (498)	79.32	79.56	0.80 (735)	80.36	0.25 (237)
Black or Black British – Caribbean	75.01	0.44 (44)	75.45	75.47	0.78 (78)	76.25	0.34 (34)
Black or Black British – any other Black background	62.82	0.66 (57)	63.48	62.79	1.43 (129)	64.22	0.77 (72)
Black or Black British – African	66.24	0.80 (227)	67.04	65.87	1.89 (575)	67.76	1.09 (348)
**Index of multiple deprivation**							
1 – Most deprived	71.76	0.64 (1,868)	72.4	71.61	1.12 (3,308)	72.73	0.48 (1,440)
2	71.51	0.55 (443)	72.06	71.69	1.20 (962)	72.89	0.65 (519)
3	69.89	0.47 (227)	70.36	69.82	0.89 (435)	70.71	0.42 (208)
4	69.64	0.51 (104)	70.15	70.26	0.68 (140)	70.94	0.17 (36)
5 – Least deprived	82.8	0.42 (40)	83.22	83.44	0.71 (67)	84.15	0.29 (27)

MMR: measles–mumps–rubella vaccination.

The ethnic groups shown are the five groups with the highest rates of measles in the outbreak, and other ethnic groups have been supressed.

The outbreak period (yellow) is inclusive of vaccines administered during both the outbreak and the subsequent MMR catch-up campaign, and the non-outbreak period (teal) covers a comparable period without either outbreak or catch-up campaign. Ethnic groups are ordered from lowest to highest vaccine coverage at the start of the outbreak period.

**Figure 5:**
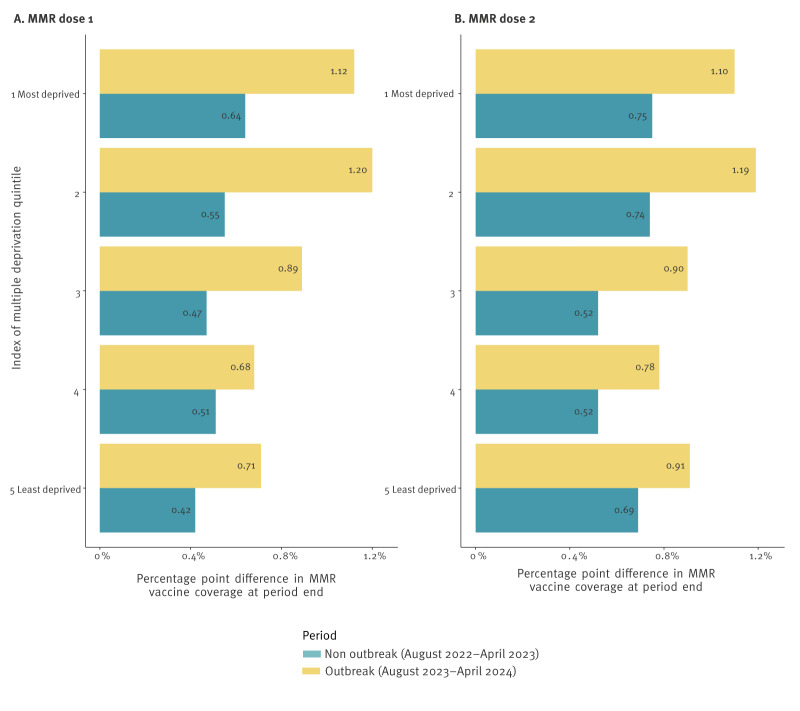
Percentage differences in MMR vaccine coverage at the end of each period compared with the start, stratified by index of multiple deprivation quintile, Birmingham, United Kingdom, 13 October 2023–12 April 2024

When comparing the change in vaccination to the same period of the previous years, a larger increase was seen for all IMD quintiles for the outbreak period (Figure 5). The largest impact was seen in IMD quintile 2, with a difference of 0.7% (an increase of 0.6% in the previous period and 1.2% in the outbreak period), and in quintile 1 with a difference of 0.5% (an increase of 0.6% in the previous period and 1.1% in the outbreak period) for MMR dose 1 (Table 3). In quintile 5, a difference of 0.3% was seen between the time periods.

MMR: measles–mumps–rubella vaccination.

The outbreak period (yellow) is inclusive of vaccines administered during both the outbreak and the subsequent MMR catch-up campaign, and the non-outbreak period (teal) covers a comparable period without either outbreak or catch-up campaign.

## Discussion

Following the introduction of measles in Birmingham, transmission initially spread through a small number of schools. A large outbreak of over 400 confirmed cases ensued, disproportionately affecting individuals living in the most deprived areas of the city. There is an intersectional relationship between deprivation and ethnicity, with these two risk factors being related, and so certain ethnic groups, including ‘Black African’ and Pakistani ethnicities also had disproportionately high case numbers and rates [25]. The rates of measles cases reported here by both ethnicity and deprivation have not been age-standardised due to lack of available denominator data and therefore may overestimate the inequalities so should be interpreted with caution. Age-stratified RRs by ethnicity showed that, when compared with the ‘White British’ population, the risk of measles in individuals up to the age of 18 years was consistently higher in other ethnic groups, and almost 30 times higher in ‘Black or Black British – Any other Black background’ teenagers. However, geographical and household clustering of these ethnic groups and of measles cases may, in part, explain the large RRs reported, and the RR estimates are not adjusted for confounders. Most cases were aged 10 years or younger, which is consistent with COVER MMR statistics which have shown a year-on-year decline in vaccination uptake in this age group between 2014 and 2024 [6]. The hospitalisation rate of 42% is higher than has been previously reported [11,15], but this may be due to case under ascertainment. Cases reported here only include those with microbiological confirmation, and not probable cases, so likely underestimate the true extent of the outbreak.

National population susceptibility estimates calculated in 2019 suggest that, outside of London, coverage is sufficient to prevent sustained outbreaks in birth cohorts born between 2008–2009 to 2013–2014 (aged 9 to 15 years old at the time of the outbreak) [5,7]. Official national COVER statistics data from 2022 to 2023 show that, before the outbreak, MMR coverage in Birmingham was below the 95% threshold required for herd immunity [4,6]. Data from COVER are known to underestimate coverage, only representing a snapshot at specific ages, and are not updated as individuals age beyond 5 years. Despite these limitations, COVER data are the most accurate, validated estimate of population coverage available, but data are published quarterly 3 months in arrears, and there is no disaggregation by ethnicity or deprivation. The IIS data from primary care vaccination records, differ from COVER data and are not validated to provide accurate population-level coverage levels, with under ascertainment known to be greater in older age groups. As a consequence, total coverage is likely to be underestimated, particularly in those over the age of 10 years. However, IIS does provide real-time, individual-level data for populations registered with a primary care doctor and therefore is a useful dataset for comparing changes in vaccination status for specific populations over time. A further limitation of IIS data is that they only include those registered with primary care, and individuals from some ethnicities or socioeconomically deprived groups may be underrepresented.

Previous outbreaks in the UK, have been driven by particular under-vaccinated communities, such as Orthodox Jewish, Steiner, migrant and Traveller communities. The last large epidemic in the UK in 2012–2013, however, was driven by low vaccination rates of teenagers which resulted from poor vaccine uptake in the early 2000s due to the media attention of the since discredited paper erroneously linking MMR to autism [5,30]. The decade-long decreasing trend in MMR coverage and increasing inequality gap observed nationally and in Birmingham meant that there was an increased pool of susceptible children under the age of 10 years. In this outbreak 21% of cases (87/406) were from Pakistani communities and 14% from ‘Black African’ communities (58/406) (with high rates seen in these populations). Prior to the outbreak, ‘Black African’ communities were among the groups with the lowest MMR coverage (65.9% for dose 1 and 51.5% for dose 2). Pakistani communities were among the highest (79.6% for dose 1 and 64.4% for dose 2), although this was still well below the 95% target and the vaccination rates of 84.6% and 71.9% seen in ‘White British’ populations, for dose 1 and 2 respectively. As the Pakistani population is the second largest ethnic group in Birmingham (92,570 of 455,761 residents aged 1–25 years in Birmingham IIS data, 20.3%) [16], there were estimated to be ca 19,000 unvaccinated individuals aged 1–25 years of Pakistani ethnicity before the outbreak began (higher than the ca 18,000 estimated for unvaccinated ‘White British’ individuals aged 1–25 years).

Efforts throughout the outbreak were focussed on targeting control measures, in particular vaccination campaigns, at communities most at risk of measles. All ethnic groups (with the exception of ‘Gypsy or Irish Traveller’ communities for dose 1 and 2, and Arab communities for dose 2) saw a larger improvement in vaccination rates in the outbreak period compared with the same period in the previous years. Some of the ethnic minority groups who were most impacted by the outbreak were among the ethnic groups that saw the biggest benefit from the vaccination campaign (e.g. ‘Black African’), but other ethnicities that were impacted by the outbreak did not see such a large increase (e.g. ‘Asian Pakistani’). This perhaps highlights the challenges of mass catch-up campaigns reaching specific communities and the importance of tailoring public health approaches to meet their needs. With the available data, we were unable to evaluate the different vaccination interventions that were employed, such as general population catch-up campaigns or targeted vaccine clinics in schools. In this regard, prospective evaluations, in the future, of different interventions in isolation would be helpful to further inform strategies.

Outside outbreak periods, it is important to maintain high levels of vaccination uptake to avoid building up pools of susceptible individuals over time. This requires sustained, easy and equitable access to vaccination, especially for the most vulnerable groups. During outbreak response periods, even small increases in coverage, especially in the least vaccinated populations, can have a positive impact in underserved communities. Further qualitative work is required to understand the different drivers in vaccination uptake and barriers to accessing services across different ethnic groups.

Measles is known to disproportionately affect individuals living in more deprived areas [8], and this was evident in the outbreak reported here. The intersectional inequalities of ethnicity and deprivation [31], with geographic co-location of these sub-populations, mean that these communities are more likely to be under-vaccinated and exposed to infection within the same schools and social networks which allows for rapid spread. The largest gains in MMR coverage in the outbreak period compared with the same period in the non-outbreak year were in IMD quintiles 1 and 2. This indicates that the catch-up campaign was most effective in targeting the most deprived populations, who were at highest risk of measles due, in part, to lower vaccine uptake.

The ethnicity data available at the time of the outbreak, used to inform the outbreak response, was less complete and less granular than the data reported here, highlighting the sub-optimal availability or recording of ethnicity in healthcare data [32]. This high unrecorded rate may be a result of limitations in the way data are collected, how they are made available for analysis, lack of access to healthcare services for some underserved populations, or due to individuals purposefully withholding their ethnicity data.

## Conclusion

This outbreak has highlighted ongoing inequalities in vaccination coverage, impact of vaccine catch-up campaigns, and disproportionate risk of measles among particular ethnic groups and socioeconomically deprived populations in Birmingham. While inequalities in vaccine uptake in subpopulations exist, the potential for significant measles outbreaks remain. UKHSA and partner organisations have a strategic ambition to reduce inequalities. The UKHSA Immunisation Inequalities Strategy recommends locally driven, tailored programmes to address barriers to uptake, as championed by the WHO’S Tailoring Immunisations Programmes approach. To facilitate this, further work to improve the quality of ethnicity data for infectious disease and vaccination surveillance is required, to allow populations at highest risk to be identified. Ongoing work to target vaccination and tailor services for underserved communities, including the most deprived populations and ethnic minorities, is required and should be supported by qualitative work to understand the different challenges and barriers to vaccination across different communities.
